# Assemblage and functional categorization of dung beetles (Coleoptera: Scarabaeinae) from the Pantanal

**DOI:** 10.7717/peerj.3978

**Published:** 2017-11-08

**Authors:** Marcelo B. Pessôa, Thiago J. Izzo, Fernando Z. Vaz-de-Mello

**Affiliations:** 1 Department of Ecology/Progama de Pós Graduação em Ecologia e Evolução, Universidade Federal de Goiás, Goiânia, Brazil; 2 Department of Botany and Ecology, Universidade Federal de Mato Grosso, Cuiabá, Brazil; 3 Department of Biology and Zoology, Universidade Federal de Mato Grosso, Cuiabá, Brazil

**Keywords:** Functional groups, Functional traits, Diversity, Guilds

## Abstract

The Pantanal is one of the world’s largest tropical wetland areas and harbors high mammal biomass. There is no formal list of dung beetle species, and studies on their functional roles have never being carried out in Pantanal. In this study, we identified dung beetle species occurring in the north Pantanal region (Poconé sub-region, Brazil) and studied their functional organization, by measuring morphological, behavioral and phenological traits. We collected 25,278 individuals belonging to 17 genera and 35 species. We identified eight functional groups in the habitat: Noturnal Telecoprids, Diurnal Telecoprids, Nesting Endocoprids, Small Nonrollers, Nocturnal Nester Paracoprids, Big Nesters Paracoprids, Non Nesters Paracoprids and Diurnal Nesters Paracoprids. The functional groups were defined mostly by two reproductive traits and two niche differentiation traits related to the use of fecal resources. This high diversification of both species and functional roles shows the importance of the group in a habitat with strong variation in availability of habitat and resources.

## Introduction

Understanding the role of a species in the environment has been a challenge to ecologists for a long time. As trivial as it may seem, addressing the function of a species is not simple. For most species, natural history and functional relationships are unknown and there are few observational data addressing this issue. To solve this problem, ecologists use characteristics of species that may represent or influence their function—their functional traits. A functional trait is defined as any measurable characteristic (morphological, biochemical, phenological, physiological or behavioral) that is measured at the individual level and affects the individual’s fitness (Violle et al., 2007). In spite of its widespread use in plant functional ecology (Perez-Harguindeguy et al., 2013), the use of traits in animal functional ecology is less disseminated and is emphasized mostly in assembling processes (Moretti et al., 2017).

Dung beetles, as an exception, are one of the groups where experiments with traits have been carried out. For instance, size was observed in terms of how it affects the removal of feces (Slade et al., 2007), how it affects the secondary dispersal of seeds (Andresen, 2003), and how it responds to soil structure (Davis, 1996). Horn size was observed in terms of how it affects mating success and sexual selection (Emlen et al., 2005).

In historical dung beetle research there has been an interest in identifying the differences between species that may represent different functional effects and responses. Several authors have proposed and defined established guilds for this group analyzing different aspects of behaviors and resource use. Halffter & Mathews (1966) used nesting type to characterize and define four groups. This classification used the behavioral differences among species and provided the basis for proposing evolutionary relationships regarding nesting behaviors. Another classification was proposed by Bornemissza (1969) and later expanded by Doube (1990). This classification defined groups by their resource allocation strategies (Bornemissza, 1969): telecoprid, paracoprid, endocoprid and cleptocoprid. The telecoprids form dung balls that are rolled away, buried, and used for feeding and reproduction. The paracoprids create underground chambers directly below the feces, often built before the resource is brought down; and after being excavated they build their nests using the feces above (Bornemissza, 1969). Unlike the other groups mentioned, endocoprids and cleptocoprids do not displace or bury the resource (Doube, 1990); instead, endocoprids use the resource directly, while the cleptocoprids are parasites of dung balls or nests of paracoprids and telecocripds. Later, Doube (1990) included other features for categorization, such as the time associated with the resource allocation and size of individuals. Seven groups were then proposed: group 1—large telecoprids, group 2—small telecoprids, group 3—paracoprid fast diggers, group 4—large paracoprid slow diggers, group 5—small paracoprid slow diggers, group 6—endocoprids, and group 7—cleptocoprids.

These divisions in groups based on natural history are interesting and allow for a rapid functional analysis of community composition. However, this classification system is not precise, essentially because the criteria for the categorization of groups are subjective; there are no clear boundaries or limits for the real meaning of “fast and slow”, and no biological reasoning for the boundary of 10 mm in “large and small” categories. In addition, subjective definitions can change over time and according to the studied habitat. Moreover, the majority of neotropical species are virtually unknown—both in terms of their natural history and phylogenetic relationships—so phylogenetic generalizations, a common way to address the functionality of species in neotropical studies, are made only from the few species whose natural history is known. This is a serious problem, since the groups proposed back in the 1990’s were based on African beetles (Doube, 1990) and do not have the phylogenetic support on neotropical species (Vaz-de-Mello, 2007). Therefore, such functional generalizations based on relatedness can place animals in the wrong groups, which could lead to errors in functional analysis.

The interest in dung beetles and their role in the environment encouraged ecologists to identify their functions. The functions of scarabeids in the ecosystem are extremely diverse, including nutrient cycling, soil bioturbation, secondary dispersal of seeds, increased incorporation of nitrogen in the soil, parasite removal, control of flies, trophic regulation and pollination (Nichols et al., 2008). Because of their high local and regional diversity and their use of feces as a resource, they represent a bioindicator of mammalian diversity (Halffter & Mathews, 1966). Based on their rapid response to environmental changes (Gardner et al., 2008a) and high efficacy in monitoring such changes (Gardner et al., 2008b), these beetles are considered good bioindicators of environmental changes (g.e. forest loss, fire disturbance). Thus, dung beetles may be a focal group to understand environmental changes, especially in mammal-rich regions, because of the strong relations between dung beetles and mammals.

Because of the high mammalian abundance in the Pantanal (Junk et al., 2006) studies of diversity and functional behavior of dung beetles (Scarabeinae) may be particularly interesting. The Pantanal preserves large herbivores species such as *Tapirus terrestris* (Tapir) and *Blastocerus dichotomius* (Pantanal Deer), as well as large carnivore species such as *Panthera onca* (Jaguar), *Puma concolor* (Puma) (Alho, Camargo & Fischer, 2011). Also present are large numbers of domestic cattle and other introduced species such as *Sus scrofa* (a feral form known as porco Monteiro) and *Bubalus bubalis* (Water buffalo) (Mourão et al., 2002). Therefore, the high production of feces in this region and the seasonality imposed by the flood pulse, increases the importance of knowing the effects of dung beetles in feces removal. However, despite the high diversity of the scarabeids, few studies on the Pantanal can be found (Aidar et al., 2000; Lopes, 2000; Silva et al, 2005; Louzada, Lopes & Vaz-de Mello, 2007; Tissiani, 2009; Rodrigues et al., 2010). Upon consulting the Entomological Collection of the Federal University of Mato Grosso and collections made in other regions, more than 70 species were found (MB Pessôa and FZ Vaz-de-Mello, pers. comm., 2011), but no comprehensive list of beetle species in the Pantanal has been published.

The use of traits to determine functional groups provides important insights into the response of a community to disturbances or habitat changes (Barragán et al., 2011; Braga et al., 2013). Thus, analysis of morphological traits associated with functional characteristics of the species is an extremely useful tool to understand functional diversity, to propose new groups, or to allow the analysis of the existing groups in different biogeographical regions, under different ecological and evolutionary pressures. In this study, we carried out an inventory on dung beetles of the Pantanal region and used their morphological, behavioral, and phenological traits to identify and propose a classification of functional groups to which they belong.

## Material and Methods

### Study area

**Figure 1:**
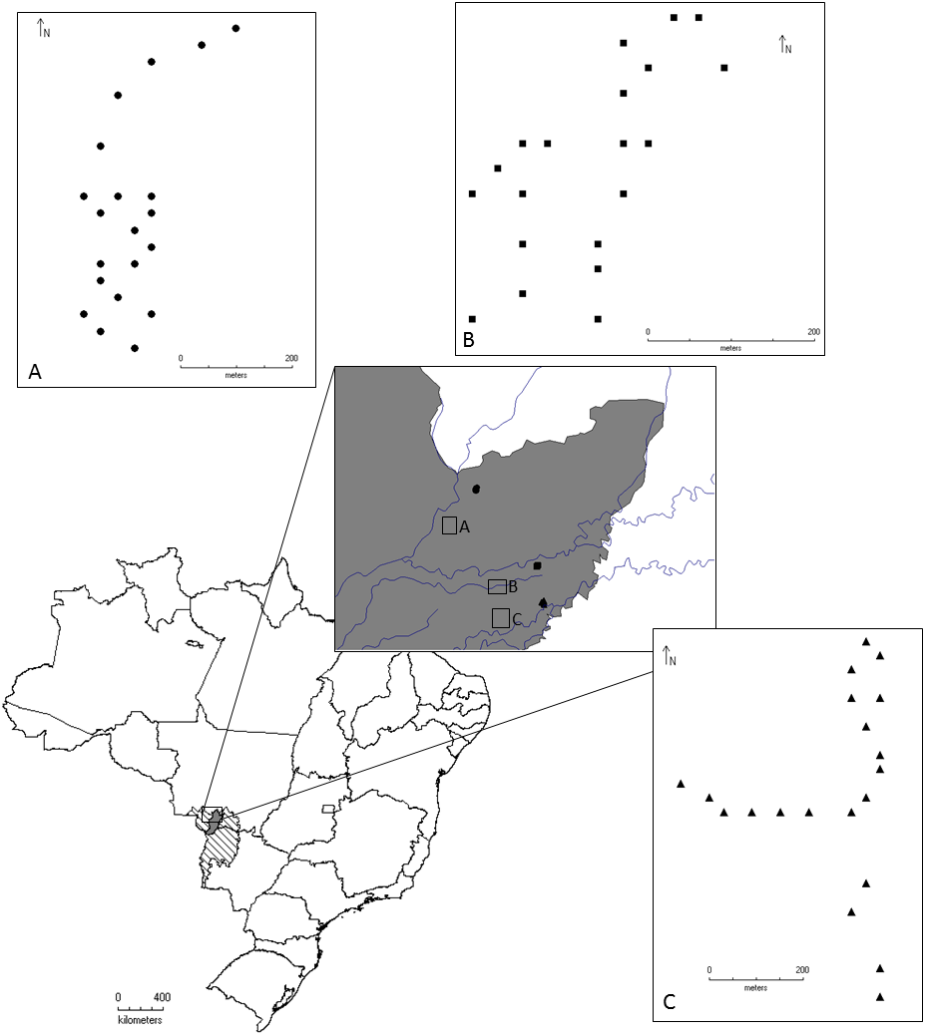
Geographical location of the localities where the beetles were collected for this study, Pantanal, Mato Grosso, Brazil. Circles represent Area 1, Conceição Farm. Squares represent Area 2, Alvorada Farm, and Triangles represent Area 3 in RPPN SESC Pantanal. Samples were collected between the months of August/2011 and May/2012.

The study was conducted in the Pantanal of Poconé, Mato Grosso, in an area located between the Bento Gomes River and the Base of Advanced Studies of the Federal University of Mato Grosso, on the property of the SESC Pantanal, near the Cuiabá River (Fig. 1).

The Brazilian Pantanal consists of vast wetlands formed by the Paraguay River floodplain and its tributaries (Mercante, Rodrigues & Ross, 2011). Wetlands are composed of heterogeneous associations of land and flooded areas and/or floodplains. These areas are responsible for a range of ecosystem services such as provisioning food and water, regulating climate and hydrological cycles, supporting biodiversity, soil formation, and nutrient cycling (Millennium Ecosystem Assessment, 2005). Because of its environmental heterogeneity, the Pantanal is divided into sub-regions (Silva & Abdon, 1998) based on topography, flooding, soil type, and vegetation. The Poconé sub-region represents 11.63% of the Pantanal area, and 52% of that sub-region is characterized as savanna, although woody savannas, grassy-woody savannas, grasslands and pioneer forested formations can also be found (Abdon & Silva, 2006). Floodplains in the Poconé sub-region can be categorized into three zones according to flood intensity: low intensity, with water depths of up to 0.5 m; medium intensity, about 0.5 to 1 m; and high intensity, greater than 1 m (Abdon & Silva, 2006).

For sampling, three large areas were defined, representing the major diversity of flooding and habitat types in the region:

Area 1: Conceição Farm (16°19′50″S, 56°30′19″W), harbors “murunduns” fields and “cordilheira vegetations” within a savanna vegetation matrix (Nunes da Cunha & Junk, 2009). This is an area with lower intensity of flooding.

Area 2: Alvorada Farm (16°26′54″S, 56°24′45″W), characterized by dry forest with monodominant formation of *Callisthene fasciculata* (Mart.) Spreng. (Nunes da Cunha & Junk, 2009). It is an area of median (intermediate) intensity of flooding.

Area 3: Baía das Pedras (16°30′11″, 56°24′05″W), is characterized by semi-deciduous forest (Nunes da Cunha & Junk, 2009). It is the area with the highest intensity of flooding. Both Conceição and Alvorada farms have livestock grazing.

### Data collection

In each area, 20 baited pitfall traps were placed 50 m apart. Traps consisted of a 1 L plastic receptacle, with an opening of 14 cm, baited with 50 g of human feces suspended directly above the pitfall with wire and a 50 ml plastic container. The traps remained at the sites for 48 h (Larsen & Forsyth, 2005). Samples were collected during all seasons, including dry (22 to 26/VII/2011), flood (16 to 23/XI/2011), full (30/III/2012 to 03/IV/2012), and draining (14 to 23/V/2012). To assess the level of specificity in food preferences, five points in each area were selected at random. At these points baited traps were used with 50 g of human feces and 50 g of rotting bovine spleen as bait. For controls we used data from collections without baits which were available in a previous study in the same area (Carneiro, 2012). Vouchers were deposited at the Entomology Section, Zoological Collection of the Biosciences Institute, Federal University of Mato Grosso (CEMT—curator FZVM). Field experiments were approved by the Instituto Chico Mendes de Conservação da Biodiversidade (ICMBIO)—Sistema de Autorização e Informação de Biodiversidade (SISBIO) 16823-1.

### Functional traits

**Figure 2:**
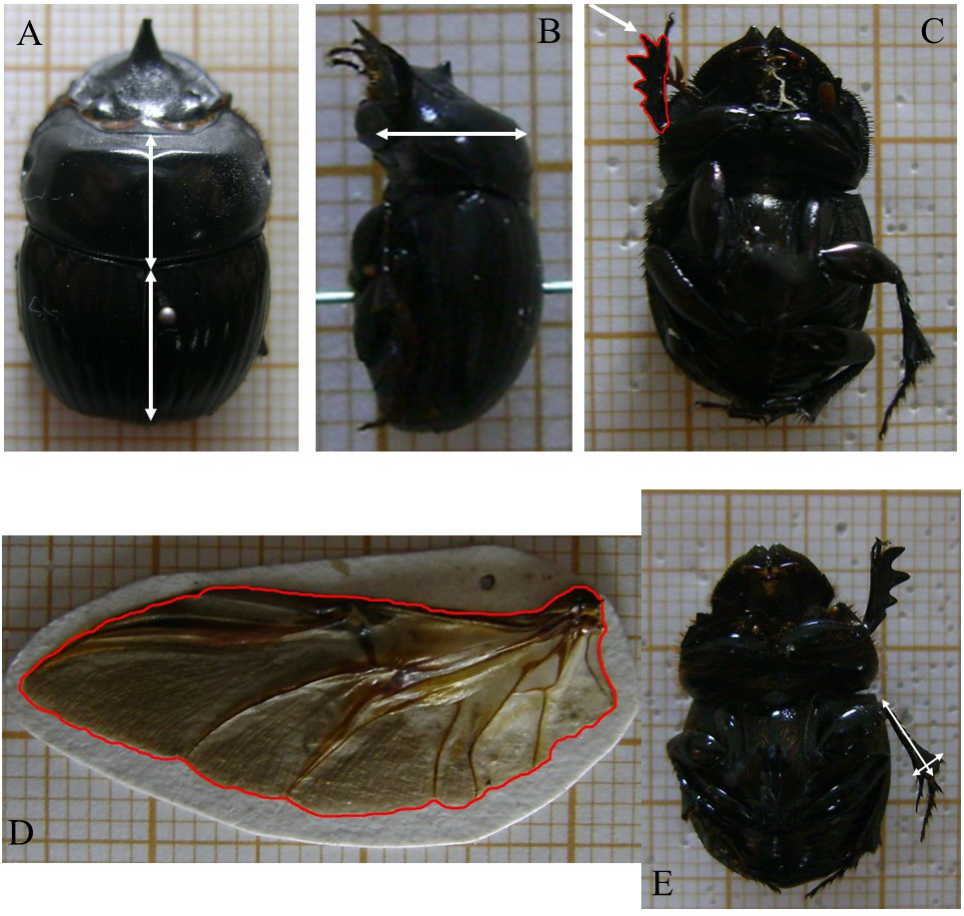
Dung beetle morphological functional trait measurements. In (A) size is measured as the sum of pronotum and elytra length. (B) Prothorax height. (C) Anterior tibiae area. (D) Wing load, measured as the ratio between wing area and size. (E) Mesotibia ratio, measured as the ratio of tibial apical width and mesotibia length.

Whenever possible, thirty individuals of each species were measured to obtain the following functional traits:

Physical Traits (Fig. 2):

(A) Size: size was defined by adding pronotal and elytra lengths to minimize variation that might occur when taking head and pigydium into account. An individual’s size impacts the amount of feces it allocates, and is a proxy for the amount of resources consumed at the larval stage for his development (Andresen, 2003; Emlen et al., 2005).
(B) Prothorax height: since the muscles of the forelegs are located in this region, individuals with greater prothorax height should have more muscle mass, suggesting a greater ability to dig (Pringle, 1939; Vilhelmsen, Miko & Krogmann, 2010).
(C) Area of the anterior tibia: We assume that an individual with the largest tibial area has more muscle capacity and consequently greater ability to dig, implying a greater ability to remove material (Halffter & Mathews, 1966).
(D) Wing load: measured by the ratio of wing area and the size/length. Based on such metric, the dispersal capability of the individual can be inferred, a higher ratio implies less effort to sustain flight. We assume that individuals with greater dispersal capability can better colonize (or recolonize) an environment (Byrne, Buchmann & Spangler, 1988; Dudley, 2002; Hongo, 2010).
(E) Mesotibia ratio: ratio of apical middle tibia width to middle tibia length. This ratio apparently separates species with rolling behavior from those having digging habits. Rolling species are usually characterized by thin tibia, while digging species have broad tibia. Species lacking these behavioral patterns can have both types of tibias (FZ Vaz-de-Mello, pers. comm., 2011).

Excluding generalism, behavioral traits were measured in binary form based on the presence (1) or absence (0) of the behavior. Except for generalism, all behaviors described here were defined by consulting literature and phylogenetic approaches. These generalizations were also made with great care, and only for behavioral characteristics. We did not use them to define *a priori* the functional groups, a practice that we previously criticized.

(A) Generalism in food preferences: species that use more than one resource should be subjected to less direct competition in unstable environments. This trait was defined by the nonstandardized method of Levin‘s niche breadth (Krebs, 1989). This index can discriminate among species that use a number of different resources, because the greater the width of the niche the greater the generalist capacity of the species (Falqueto, Vaz-de-Mello & Schoereder, 2005). There were no categorization of the trait.
(B) Horizontal displacement: horizontal displacement behavior involves moving the resource to be used away from its source. This displacement activity differs from excavation, and it allows the beetle to reduce the intensity of competition by moving a resource in the form of a ball away from the fecal deposit (Halffter & Mathews, 1966).
(C) Nest building: some species of dung beetles show complex parental care, making it possible for larvae to develop in otherwise unstable environments (Halffter & Mathews, 1982).
(D) Ball or pear-shaped nest: when building the nest, some species create ball or pear-shaped structures for larvae development, creating a more favorable environment (Halffter & Mathews, 1982).

Phenological trait: phenological traits were obtained from literature, classified as nocturnal, diurnal or mixed.

(A) Daily Activity: temporal variation is considered a mechanism of ecological segregation between potentially competing species (Feer & Pincebourde, 2005; Hernández, 2002).

### Functional categorization

We calculated the dissimilarity of the measured traits among studied species using the Gower index. To determine the functional categories, we used a non-hierarchical K-Means clustering method. The k-Means method produces different “k” sets with the highest distinction possible between them. This method is used to determine *a priori* the number of groups to be found. To determine this number, we used randomizations of 100 initial group numbers, and to define the best partition we used Calinski’s criteria (Calinski & Harabasz, 1974). These criteria define the partitions/divisions as groups that had less variation between the species that comprise the same group (within group variation) than the variation between groups (Calinski & Harabasz, 1974). Each partition that was obtained was considered as a functional group.

**Table 1:** Individuals of Scarabaeinae subfamily beetles collected in three different areas of Pantanal subregion of Poconé, Mato Grosso, Brazil.

Species	Area 1		Area 2		Area 3		Total
	FL	NF	FL	NF	FL	NF	
*Anisocanthon aff*. *villosus* (Harold, 1868)	–	–	–	–	4	–	4
*Ateuchus carbonarius* (Harold, 1868)	81	479	–	–	–	–	560
*Ateuchus* sp.1	117	972	224	39	89	162	1,603
*Canthidium viride* (Lucas, 1859)	44	100	1	1	–	–	146
*Canthidium barbacenicum* Preudhomme de Borre, 1886	277	1,839	8	2	75	26	2,227
*Canthidium cuprinum* Harold, 1867	39	90	743	149	2,161	4,243	7,425
*Canthidium* sp.2	–	–	30	5	–	–	35
*Canthidium* sp.3	–	1	–	–	–	–	1
*Canthon histrio* (LePeletier de Saint-Fargeau & Audinet-Serville, 1828)	88	94	7	9	2	–	200
*Canthon lituratus* (Germar, 1813)	12	68	–	–	1	1	82
*Canthon maldonadoi* Martinez, 1951	3	–	–	4	1	11	19
*Canthon curvodilatatus* Schmidt, 1922	10	8	3	5	4	2	32
*Canthon aff. ornatus* Redtenbacher, 1868	–	1	–	–	–	–	1
*Canthon quinquemaculatus* Castelnau, 1840	–	–	53	38	187	247	525
*Canthon daguerrei* Martínez, 1951	229	2	607	652	1	4	1,495
*Coprophanaeus bonariensis* (Gory, 1844)	–	3	–	–	–	–	3
*Coprophanaeus milon* (Blanchard, 1846)	–	6	7	15	–	–	28
*Deltochilum elongatum* Felsche, 1907	12	1	–	–	–	–	13
*Dichotomius bos* (Blanchard, 1846)	59	203	13	26	1	–	302
*Dichotomius lycas* (Felsche, 1901)	5	36	–	–	–	–	41
*Dichotomius nisus* (Olivier, 1789)	92	90	1	4	–	–	187
*Dichotomius opacipennis* (Luederwaldt, 1931)	24	253	–	–	–	–	277
*Digitonthophagus* sp. (Fabricius, 1787)	4	–	–	5	–	–	9
*Eurysternus caribaeus* (Herbst, 1789)	3	31	121	93	338	846	1,432
*Eurysternus nigrovirens* Génier, 2009	–	–	1	1	53	301	356
*Genieridium cryptops* (Arrow, 1913)	1	33	–	–	–	–	34
*Gromphas inermis* Harold, 1869	–	–	–	1	–	–	1
*Malagoniella aff. astyanax* (Olivier, 1789)	10	133	–	1	1	–	145
*Ontherus appendiculatus* (Mannerheim, 1829)	134	661	30	61	–	8	894
*Ontherus digitatus* Harold, 1868	1	30	–	1	–	–	32
*Ontherus sulcator* (Fabricius, 1775)	9	30	749	1018	519	1,254	3,579
*Onthophagus* aff. *hirculus* Mannerheim, 1829	58	230	90	180	29	36	623
*Trichillidium quadridens* (Arrow, 1932)	72	169	93	78	127	424	963
*Trichillum externepunctatum* Preudhomme de Borre, 1880	86	8	2	5	–	–	101
*Uroxys aff*. *corporaali* Balthasar, 1940	31	1,491	26	16	7	5	1,576

**Notes.**

FL floodable environment NF nonfloodable environment Area 1 Conceição Farm Area 2 Alvorada Farm Area 3 RPPN SESC Pantanal Baia das Pedras

Collected between August 2011 and May 2012.

## Results

### Species composition

We collected 25,278 individuals (Table 1) belonging to 17 genera and 35 species. The five most abundant species were *Canthidium cuprinum* (39.90%), *Ontherus sulcator* (14.24%), *Canthidium barbacenicum* (8.88%), *Ateuchus* sp. (6.46%) and *Uroxys aff*. *corporaali* (6.31%), which together accounted for 65.78% of the total sample. In Area 1, the most abundant species were *C*. *barbacenicum* (24.63%), *U. aff*. *corporaali* (17.79%), *Ateuchus* sp. (12.92%), *Ontherus appendiculatus* (23.9%) and *Ateuchus carbonarius* (6.58%), which accounted for 71.15% of the total sample. In Area 2, the most abundant species were *O*. *sulcator* (33.58%), *Canthon daguerrei* (23.88%), and *Canthidium cuprinum* (17.86%), which together accounted for 75.32% of the total sample. In Area 3, the most abundant species were *Canthidium cuprinum* (57.33%), *O*. *sulcator* (15.71%), and *Eurysternus caribaeus* (10.77%), which together represented 83.81% of the sample.

### Functional categorization

**Figure 3:**
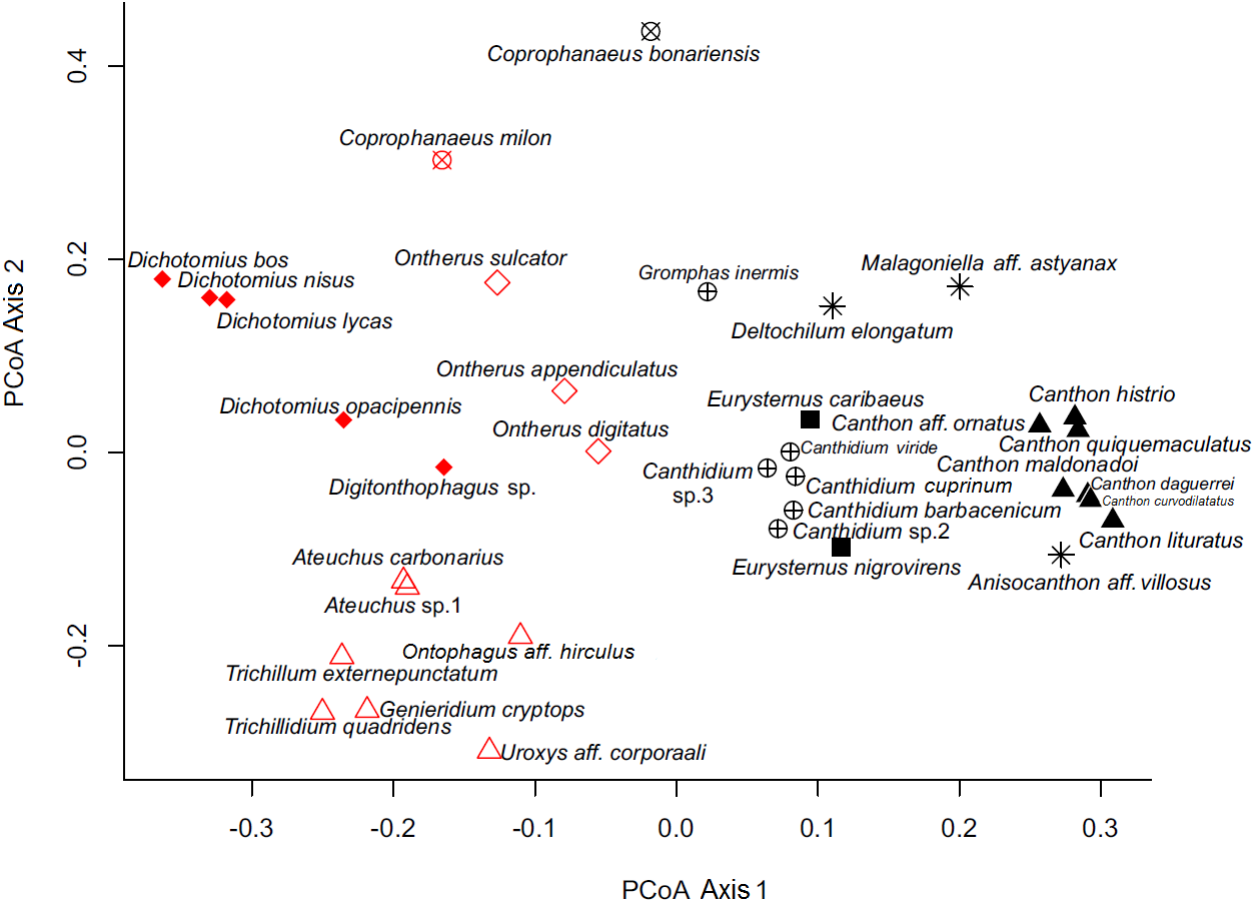
Ordination of dung beetle species (Coleoptera; Scarabaeinae), collected in the sub-region Poconé, Pantanal, Mato Grosso, Brazil, by PCoA with Gower dissimilarity index using the functional matrix, where the colors and the symbols represent the groups identified by the k-means. Colors represent the groups of the first partition/division, where the color black represents group A; the color red represents group B. Symbols represent the groups of the second partition, where: hollow diamond = group 1, asterisk = group 2, filled square = group 3, hollow triangle = group 4, filled triangle = group 5, x circle = group 6, filled diamond = group 7 and cross circle = group 8.

By analyzing the partitions/divisions obtained by randomizations we obtained two partitions/divisions of interest. The first partition/division consists of two functional groups (Table 2) and the second consists of eight functional groups (Fig. 3). The first partition/division is formed by a group of species which forms a ball or pear-shaped nest, have mainly diurnal activity and, when nocturnal, present horizontal displacement of dung behavior. The second group is comprised of species that do not form a ball or pear-shaped nest, and have nocturnal or mixed activity.

**Table 2:** The two groups of first partition/division were formed by k-means composed from 100 random partitions/divisions, using the Gower dissimilarity index, with an *a posteriori* analysis of the groups for a community of dung beetles (Coleoptera: Scarabaeinae) collected in the Pantanal subregion of Poconé, Mato Grosso, Brazil.

Group	Group characteristics	Species
A	Diurnal-mixed activity, make ball/pear-shaped nest, when nocturnal displaces horizontally the resource	*Anisocanthon aff. villosus*, *Deltochilum elongatum*, *Malagoniella aff. astynanax, Eurysternus caribaeus*, *Eurysternus nigrovirens*, *Canthon daguerrei*, *Canthon histrio*, *Canthon lituratus*, *Canthon maldonadoi*, *Canthon curvodilatatus*, *Canthon aff. ornatus*, *Canthon quinquemaculatus*, *Canthidium viride*, *Canthidium barbacenicum*, *Canthidium cuprinum*, *Canthidium* sp.2, *Canthidium* sp.3, *Gromphas inermis, Coprophanaeus bonariensis*
B	Nocturnal-mixed activity, does not make ball/pear-shaped nest, when making a ball/pear-shaped nest, it does not displace horizontally to the resource	*Coprophanaeus milon*, *Ontherus appendiculatus*, *Ontherus digitatus*, *Ontherus sulcator*, *Ateuchus carbonarius*, *Ateuchus* sp.1, *Genieridium cryptops*, *Onthophagus aff. hirculus*, *Trichillidium quadridens*, *Trichillum externepunctatum*, *Uroxys aff. corporaali*, *Dichotomius bos*, *Dichotomius lycas*, *Dichotomius nisus*, *Dichotomius opacipennis*, *Digitonthophagus sp.*

The second partition/division separated eight different groups and was characterized as follows:

Group 1—Nocturnal telecoprids: species that move the resource horizontally and are nocturnal. Species in this group are *Anisocanthon aff*. *vilosus*, *Deltochilum elongatum*, and *Malagoniella aff. astynanax*.
Group 2—Diurnal telecoprids: Diurnal species that move the resource horizontally. Species in this group are *Canthon daguerrei*, *Ca. histrio*, *Ca. lituratus*, *Ca. maldonadoi*, *Ca. curvodilatatus*, *Ca. ornatus*, and *Ca. quinquemaculatus*.
Group 3—Nesting endocoprids: species that do not displace the resource horizontally, do build ball or pear-shaped nest, are smaller than 15 mm, and can be diurnal and nocturnal. Species in this group are *Eurysternus caribaeus* and *E. nigrovirens*.
Group 4—Small non-rollers: species that do not displace the resource horizontally, do not build ball or pear-shaped nest and are smaller than 6 mm. Species in this group are *Ateuchus carbonarius*, *A.* sp.1, *Genieridium cryptops*, *Onthophagus aff*. *hirculus*, *Trichillidium quadridens*, *Trichillum externepunctatum*, and *Uroxys aff*. *corporaali*.
Group 5— Nocturnal nesting paracoprids: species that do not horizontally displace the resource, do build a ball or pear-shaped nest, are smaller than 15 mm, and are nocturnal. Species in this group are *Ontherus appendiculatus*, *O. digitatus*, and *O. sulcator*.
Group 6—Large nesting paracoprids: species that do not displace the resource horizontally, do build ball or pear-shaped nest, and are larger than 15 mm. This group consists of *Coprophanaeus bonariensis* and *Co. milon*.
Group 7—Non-nesting paracoprids: species larger than 6 mm that do not displace the resource horizontally and do not build ball or pear-shaped nest. Species in this group are *Dichotomius bos*, *D. lycas*, *D. nisus*, *D. opacipennis*, and *Digitonthophagus* sp.
Group 8—Diurnal nesting paracoprids: species that do not displace the resource horizontally, do build ball or pear-shaped nest, are smaller than 15 mm, and are diurnal. Species in this group are *Canthidium viride*, *C. barbacenicum*, including *C.  cuprinum C.* sp.2 *C.* sp.3, and *Gromphas inermis*.

## Discussion

### Composition remarks on Pantanal dung beetles

Among the species found in common with other studies, *D. bos*, *D. nisus*, and *Ca. lituratus* were collected in flooded environments (Silva et al, 2005; Rodrigues et al., 2010), and *C. barbacenicum* was found in dry areas (Louzada, Lopes & Vaz-de Mello, 2007; Rodrigues et al., 2010; Lopes, 2000). *Trichillum externepunctatum* was found in both flooded (Lopes, 2000) and dry areas (Louzada, Lopes & Vaz-de Mello, 2007; Rodrigues et al., 2010). We emphasize the presence, even relatively rare, of *Digitonthophagus* sp. in the study area. This is an invasive species introduced to Brazil in order to control the incidence of horn flies (Miranda, Nascimento & Bianchin, 1990).

*Eurysternus caribaeus*, *Ateuchus* sp., *O. aff*. *hirculus*, *C. barbacenicum*, *C. cuprinum*, *O. sulcator* and *T. quadridens* occurred in all studied areas. *Dichotomius bos* and *D.* *nisus* are species commonly found in grasslands and savannas (Louzada, Lopes & Vaz-de Mello, 2007) and in this study both occurred in savanna and dry forest. *Trichillum externepuncatatum* and *O. appendiculatus* occur throughout most of South America (Vaz-de-Mello, 2008; Génier, 1996) and were abundant in all three sampled areas. The genus *Canthon* Hoffmansegg, 1817 is comprised of several species and is only found in the New World. Most of the species are Neotropical (Vaz-de-Mello, 1999). Of note is the first record of *O. digitatus* in the Pantanal.

Although most of the species that we found have a wide distribution (data from Edmonds, 1994; Génier, 1996; Louzada, Lopes & Vaz-de Mello, 2007; Vaz-de-Mello, 2008; Génier, 2009; Edmonds & Zidek, 2010), the community of Pantanal dung beetles is composed mainly of species that occur in the Chaco and the Cerrado biogeographical provinces, and are usually the most common species in such provinces. This result contrasts with the biogeographical regionalization of South America proposed by Morrone (2001) who pointed out that the Pantanal is a province of the Amazonian subregion. Since the Pantanal is a relatively new formation and floods in the area impose a possible restriction for these beetles, the area is mostly colonized by species from nearby areas. Indeed, the areas that surround the Pantanal are mostly Cerrado and Chaco, with some subregions of the Pantanal with Amazonian and Atlantic Forest influences (Abdon & Silva, 2006).

### Functional biodiversity of Pantanal dung beetles

The characterization of dung beetles in two functional classifications represent the high redundancy found in this group, since the sub-family is mostly dung generalist, but it also shows that their functional biodiversity can be analyzed with more precision, reflecting the diversity of dung manipulation of these beetles.

In our functional classification, the two partitions/divisions proved robust and consistent classifications, since only one group from the second partition/division (large nesting paracoprids) was divided in the first classification. *Coprophanaeus bonariensis* and *C*. *milon* are crepuscular, but since the collection of *C*. *bonariensis* in mistnets for bird capture (FZ Vaz-de-Mello, pers. comm., 2011) occurred only in the daytime, we categorize this species as diurnal. In the first partition/division proposed here, the period of activity is an important feature in the separation of the groups. The influence of activity period and formation of ball or pear-shaped nest were also determinants in the first classification.

Parental care in dung beetles is a feature that is linked to adaptive and evolutionary success (Halffter & Edmonds, 1982). This behavior is clear by the care taken in nest building and the preparation of fecal mass (Halffter & Edmonds, 1982). Thus, in the first partition, it is evident that the nest, more specifically the type of nest built, is a functionally important characteristic that divides the community into two groups, together with the period of activity. Given the ephemeral resource used by these beetles, the intense competition apparently contributed to the evolution of the flight behavior of beetles, where each species flies for a limited, and often different, time of day (Caveney, Scholtz & Mcintyre, 1995). Thus, the temporal segregation of the community is an important factor for the coexistence of species of beetles and is mainly directed by interspecific competition (Feer & Pincebourde, 2005).

This classification into two groups, A and B, described above, is intriguing because of its phylogenetic determination, as only the genus *Coprophanaeus* is divided between the two groups. This genus is represented in this work by two species that were included in a recent review, being divided into two different subgenera (Edmonds & Zidek, 2010). *Coprophanaeus bonariensis* is inserted into the subgenus *Megaphanaeus* Olsoufieff, 1924, and *C*. *milon* is inserted into the subgenus *Coprophanaeus* Olsoufieff, 1924 (Edmonds & Zidek, 2010). Therefore, this classification is highly probable.

In the second partition/division, which forms eight groups, the classification seems to be an intermediate proposition between the classifications proposed by Doube (1990) and Halffter & Mathews (1966), although we did not employ the same traits used by these authors. Doube did not consider attributes of burrowing; and nesting, which was very well detailed by Halffter & Mathews (1966), was less detailed by the lack of available information. It is interesting to note that the period of activity and size are traits that functionally define the community as well, and demonstrate the clear division of functions between diurnal and nocturnal species (Krell et al., 2003).

Two traits are related to reproductive success, and two traits are related to niche differentiation. Both cases can be attributed to competition for scarce resources (Simmons & Ridsdill-Smith, 2011). The traits used and the groups created in the second partition allow us to functionally analyze the community, and suggest the presence of phylogenetic inertia since the grouping conserved the taxonomic classfication. However, this inertia is somehow expected, considering that the same morphological traits that functionally influence the community are used in phylogenetic studies.

Ecologically, the groups show significant relationships. Six of the eight groups (group 3, 4, 5, 6, 7 and 8) do not exhibit horizontal displacement behavior. Two of these groups (group 3 and 4) are fully or partially comprised of endocoprids (Vaz-de-Mello, 2008; Génier, 2009), exploring resources directly at the source. Therefore, half of the groups dig and bury the resource close to the source. This fact is relevant since in the absence of large paracoprid individuals there is an approximate 75% reduction in the removal of feces (Slade et al., 2007), largely affecting the functional role of the community. Thus, the diversity of groups that possess digging behavior may be an important feature of dung beetle communities to maintain this functional role. This can be observed by the high abundance of the other groups (5, 7 and 8) in the absence of group 6. Group 6, consists of species with larger individuals and is less abundant than the groups with species consisting of smaller individuals, thus being more susceptible to extinction (Larsen, Lopera & Forsyth, 2008). Larger individuals require a greater amount of resources to develop. As such, they are more susceptible to conditions that might change resource availability. This is important in a region where during the flooding cycles, availability of feces areas is restricted to dry areas diminishing the availability of resources and presenting a constraint for larger species. In all areas the groups with small individuals (group 5, 7 and 8) have higher abundance, compensating for services provided by other groups. This compensation is dependent on the degree of isolation of communities and environmental quality (Larsen, Lopera & Forsyth, 2008).

Although speculative, our data suggest that flood-pulse may affect dung beetle community dynamics, since a group is present only in the area with lower flooding and there is a simplification of communities in areas with higher flooding. The intensity of flooding may be a factor for further studies involving dung beetles, since it may affect the availability of resources on non-flooded areas and consequently alter the competitive interactions. In another way, the flooded areas may experience constant recolonization. During floods, non-flooded areas act as islands providing refuge to mammals (Alho, Camargo & Fischer, 2011). We imagine two possible scenarios: more accessible islands may have an increase in the density of resources for dung beetles and diminsh competition among dung beetles, whereas less-accessible islands may see a reduction in density of resources which would increase the competition. These different scenarios may present constraints for different functional traits altering the functional space of dung beetle community. Consequently studies on flooding and assembly rules are interesting topics to address in future functional studies. We highlight that the classification proposed here does not use any subjective criteria for functional classifications and we suggest increasing the number of species to further analyze the functional grouping of dung beetles.

##  Supplemental Information

10.7717/peerj.3978/supp-1 Supplemental Information 1 Total abundance of dung beetles captured and vouxered

10.7717/peerj.3978/supp-2 Supplemental Information 2 Individuals measured

10.7717/peerj.3978/supp-3 Supplemental Information 3 Functional matrix utilized in the study

## Additional Information and Declarations

### Field Study Permissions

The following information was supplied relating to field study approvals (i.e., approving body and any reference numbers): Field experiments were approved by the Instituto Chico Mendes de Conservação da Biodiversidade (ICMBIO)—Sistema de Autorização e Informação de Biodiversidade (SISBIO) 16823-1.

### Data Availability

The following information was supplied regarding data availability: The raw data has been supplied as a Supplemental File.

## References

[ref-1] Abdon MM, Silva JSV (2006). Fisionomias da vegetação nas sub-regiões do Pantanal [Physiognomies of vegetation in Pantanal subregions].

[ref-2] Aidar T, Koller WW, Rodrigues SR, Corrêa AM, Silva JCC, Balta OS, Oliveira JM, Oliveira VL (2000). Besouros coprófagos (Coleoptera: Scarabaeidae) coletados em Aquidauana, Ms. Brasil [Copper beetles (Coleoptera: Scarabaeidae) collected in Aquidauana, Ms. Brasil]. Anais da Sociedade Entomológica do Brasil.

[ref-3] Alho CJR, Camargo G, Fischer E (2011). Terrestrial and aquatic mammals of the Pantanal. Brazilian Journal of Biology.

[ref-4] Andresen E (2003). Effect of forest fragmentation on dung beetle communities and functional consequences for plant regeneration. Ecography.

[ref-5] Barragán NF, Moreno CE, Escobar F, Halffter G, Navarrete D (2011). Negative impacts of human land use on dung beetle functional diversity. PLOS ONE.

[ref-6] Bornemissza GF (1969). A new type of brood care observed in the dung beetle Oniticellus cinctus (Scarabaeidae). Pedobiologia.

[ref-7] Braga RF, Korasaki V, Andresen E, Louzada J (2013). Dung beetle community and functions along a habitat-disturbance gradient in the amazon: a rapid assessment of ecological functions associated to biodiversity. PLOS ONE.

[ref-8] Byrne DN, Buchmann SL, Spangler HG (1988). Relationship between wing loading, wingbeat frequency and body mass in homopterous insects. Journal of Experimental Biology.

[ref-9] Calinski RB, Harabasz J (1974). A dendrite method for cluster analysis. Communications in Statistics-Theory and Methods.

[ref-10] Carneiro AC (2012). Efeito da inundação sobre a estrutura da comunidade terrestre de Coleoptera (Arthropoda, Insecta): estudo comparativo entre áreas inundáveis e não inundáveis no Pantanal de Poconé—Mato grosso [Effect of flood on the structure of the terrestrial community of Coleoptera (Arthropoda, Insecta): a comparative study between flooded areas and not flooded in the Pantanal de Poconé—Mato Grosso]. Master’s dissertation.

[ref-11] Caveney S, Scholtz CH, Mcintyre P (1995). Patterns of daily flight activity in onitine dung beetles (Scarabaeinae: Onitini). Oecologia.

[ref-12] Davis ALV (1996). Community organization of dung beetles (Coleoptera: Scarabaeidae): differences in body size and functional group structure between habitats. African Journal of Ecology.

[ref-13] Doube BM (1990). A functional classification for analysis of the structure of dung beetle assemblages. Ecological Entomology.

[ref-14] Dudley R (2002). Mechanisms and implications of animal flight maneuverability. Integregrative and Comparative Biology.

[ref-15] Edmonds WD (1994). Revision of Phanaeus Macleay, a New World genus of Scarabaeine dung beetles (Coleoptera, Scarabaeinae). Revisión de Phanaeus Macleay, un género del Nuevo Mundo de escarabajos estercoleros (Coleoptera, Scarabaeinae). Contributions in Science.

[ref-16] Edmonds WD, Zidek J (2010). A taxonomic review of the neotropical genus *Coprophanaeus* Olsoufieff, 1924 (Coleoptera: Scarabaeidae, Scarabaeinae). Insecta Mundi.

[ref-17] Emlen DJ, Marangelo J, Ball B, Cunningham CW (2005). Diversity in the weapons of sexual selection: horn evolution in the beetle genus Onthophagus (Coleoptera: Scarabaeidae). Evolution.

[ref-18] Falqueto SA, Vaz-de-Mello FZ, Schoereder JH (2005). Are fungivorous Scarabaeidae less specialist?. Ecología Austral.

[ref-19] Feer F, Pincebourde S (2005). Diel flight activity and ecological segregation within an assemblage of tropical forest dung and carrion beetles. Journal of Tropical Ecology.

[ref-20] Gardner TA, Barlow J, Araujo IS, Avilla-Pires TC, Bonaldo AB, Costa JE, Esposito MC, Ferreira LV, Hawes J, Hernandez MIM, Hoogmoed MS, Leite RN, Lo-Man-Hung NF, Malcom JR, Martins MB, Mestre LAM, Miranda-Santos R, Overal WL, Parry L, Peters SL, Ribeiro-Junior MA, Silva MNFda, Motta CSda, Peres CA (2008b). The cost-effectiveness of biodiversity surveys in tropical forests. Ecology Letters.

[ref-21] Gardner TA, Hernández MIM, Barlow J, Peres CA (2008a). Understanding the biodiversity consequences of habitat change: the value of secondary and plantation forests for neotropical dung beetles. Journal of Applied Ecology.

[ref-22] Génier F (1996). A revision of the Neotropical genus Ontherus Erichson. Memoirs of the Entomological Society of Canada.

[ref-23] Génier F (2009). Le genre Eurysternus Dalman, 1824 (Scarabaeidae: Scarabaeinae: Oniticellini), revision taxonomique et clés de determination illustrées [The genus Eurysternus Dalman, 1824 (Scarabaeidae: Scarabaeinae: Oniticellini), taxonomic revision and determination keys illustrated].

[ref-24] Halffter G, Edmonds WD (1982). The nesting behavior of dung beetles (Scarabaeinae). An ecological and evolutive approach.

[ref-25] Halffter G, Mathews EG (1966). The natural history of dung beetles of the subfamily Scarabaeinae (Coleoptera, Scarabaeidae). Folia Entomologica Mexicana.

[ref-26] Hernández MIM (2002). The night and day of dung beetles (Coleoptera, Scarabaeidae) in the Serra do Japi, Brazil: elytra colour related to daily activity. Revista Brasileira de Entomologia.

[ref-27] Hongo Y (2010). Does flight ability differ among male morphs of the Japanese horned beetle Trypoxylus dichotomus septentrionalis (Coleoptera Scarabaeidae)?. Ethology Ecology & Evolution.

[ref-28] Junk WJ, Nunes-Da-Cunha C, Wantzen KM, Petermann P, Strüssmann C, Marques MI, Adis J (2006). Biodiversity and its conservation in the Pantanal of Mato Grosso, Brazil. Aquatic Sciences.

[ref-29] Krebs CJ (1989). Ecological methodology.

[ref-30] Krell F-T, Krell-Westerwalbesloh S, Weiß I, Eggleton P, Linsenmair KE (2003). Spatial separation of Afrotropical dung beetle guilds: a trade-off between competitive superiority and energetic constraints (Coleoptera: Scarabaeidae). Ecography.

[ref-31] Larsen TH, Forsyth A (2005). Trap spacing and transect design for dung beetle biodiversity studies. Biotropica.

[ref-32] Larsen TH, Lopera A, Forsyth A (2008). Understanding trait-dependent community disassembly: dung beetles, density functions and forest fragmentation. Conservation Biology.

[ref-33] Lopes VA (2000). Comunidades de Scarabaeidae Stricto sensu (Coleoptera) em quatro tipos de vegetação nativa do Pantanal Sul-mato-grossense [Communities of Scarabaeidae Stricto sensu (Coleoptera) in four types of vegetation native to Pantanal Sul-mato-grossense]. Master’s dissertation.

[ref-34] Louzada JNC, Lopes FS, Vaz-de Mello FZ (2007). Structure and composition of a dung beetle community (Coleoptera, Scarabaeinae) in a small Forest patch from the Brazilian Pantanal. Revista Brasileira de Zoociências.

[ref-35] Mercante MA, Rodrigues SC, Ross JLS (2011). Geomorphology and habitat diversity in the Pantanal. Brazilian Journal of Biology.

[ref-36] Millennium Ecosystem Assessment (2005). Ecosystems and human well-being: wetlands and water *synthesis*.

[ref-37] Miranda CHB, Nascimento YA, Bianchin I (1990). Desenvolvimento de um programa integrado de controle dos nematódeos e a mosca-dos-chifres na região dos Cerrados [Development of a program integrated control of nematodes and the horn-fly in the region of Cerrados]. Fase 3. Potencial De Onthophagus Gazella No Enterrio De Fezes Bovinas.

[ref-38] Moretti M, Dias ATC, De Bello F, Altermatt F, Chown SL, Azcárate FM, Bell JR, Fournier B, Hedde M, Hortal J, Ibanez S, Öckinger E, Sousa JP, Ellers J, Berg MP (2017). Handbook of protocols for standardized measurement of terrestrial invertebrate functional traits. Functional Ecology.

[ref-39] Morrone JJ (2001). Biogeografía de América Latina y el Caribe.

[ref-40] Mourão DM, Coutinho ME, Mauro RDA, Tomás WM, Magnusson W (2002). Levantamentos aéreos de espécies introduzidas no Pantanal: porcos ferais (porco monteiro), gado bovino e búfalos [Withdrawals of species introduced into the Pantanal: feral pigs (monkey pig), cattle and buffalo]. Embrapa Pantanal-Boletim de Pesquisa e Desenvolvimento (Infoteca-E).

[ref-41] Nichols E, Spector S, Louzada J, Larsen T, Amezquita S, Favila ME, The Scarabaeinae Research Network (2008). Ecological functions and ecosystems services provided by Scarabaeinae dung beetles. Biological Conservation.

[ref-42] Nunes da Cunha C, Junk WJ, Junk WJ, Da Silva CJ, Nunes da Cunha C, Wantzen KM (2009). Landscape units of the Pantanal: structure, function, and human use. The pantanal: ecology, biodiversity and sustainable management of a large neotropical seasonal wetland.

[ref-43] Perez-Harguindeguy N, Diaz S, Garnier E, Lavorel S, Poorter H, Jaureguiberry P, Urcelay C (2013). New handbook for standardised measurement of plant functional traits worldwide. Australian Journal of Botany.

[ref-44] Pringle JWS (1939). The motor mechanism of the insect leg. Journal of Experimental Biology.

[ref-45] Rodrigues SR, Barros ATM, Puker A, Taira TL (2010). Diversity of coprophagous scarab beteles (Coleoptera, Scarabaeidae) collected with flight intercept trap in the Southern Pantanal, Brazil. Biota Neotropica.

[ref-46] Silva PHda, Guedes JM, Machado AKDeFM, Vieira LM (2005). Distribuição de Scarabaeidae Stricto sensu (Coleoptera) em micro-ambientes no Pantanal Sul [Distribution of Scarabaeidae Stricto sensu (Coleoptera) in micro environments in the Pantanal Sul].

[ref-47] Silva JSV, Abdon MM (1998). Delimitação do Pantanal brasileiro e suas sub-regiões [Delimitation of the Brazilian Pantanal and its subregions]. Pesquisa Agropecuária Brasileira.

[ref-48] Simmons LW, Ridsdill-Smith TJ (2011). Ecology and evolution of dung beetles.

[ref-49] Slade EM, Mann DJ, Villanueva JF, Lewis OT (2007). Experimental evidence for the effects of dung beetle functional group richness and composition on ecosystem function in a tropical forest. Journal of Animal Ecology.

[ref-50] Tissiani ASO (2009). Composição da comunidade de Scarabaeidae coprófagos (Insecta, Coleoptera), em uma área na região norte do Pantanal de Mato Grosso, Brasil. Master’s dissertation (Ecologia e Conservação da Biodiversidade).

[ref-51] Vaz-de-Mello FZ (1999). Scarabaeidae sensu strictu (Coleoptera: Scarabaeoidea) de um fragmento de floresta amazônica no estado do Acre, Brasil [Scarabaeidae sensu strictu (Coleoptera: Scarabaeoidea) from a fragment of Amazon forest in the state of Acre, Brazil]. 1. Taxocenose. Anais da Sociedade Entomológica do Brasil.

[ref-52] Vaz-de-Mello FZ (2007). Revisión Taxonómica y Análisis Filogenético de la Tribu Ateuchini (Coleoptera: Scarabaeidae: Scarabaeinae) [Taxonomic Review and Phylogenetic Analysis of the Tribe Ateuchini (Coleoptera: Scarabaeidae: Scarabaeinae)]. Doctoral thesis.

[ref-53] Vaz-de-Mello FZ (2008). Synopsis of the new subtribe Scatimina (Coleoptera: Scarabaeidae: Scarabaeinae: Ateuchini), with descriptions of twelve new genera and review of Genieridium, new genus. Zootaxa.

[ref-54] Vilhelmsen L, Miko I, Krogmann L (2010). Beyond the wasp-waist: structural diversity and phylogenetic significance of the mesosoma in apocritan wasps (Insecta: Hymenoptera). Zoological Journal of the Linnean Society.

[ref-55] Violle C, Navas ML, Vile D, Kazakou E, Fortunel C, Hummel I, Garnier E (2007). Let the concept of trait be functional!. Oikos.

